# CRISPR/Cas Technology for the Diagnosis of Animal Infectious Diseases

**DOI:** 10.3390/microorganisms13092006

**Published:** 2025-08-28

**Authors:** Shuling Meng, Zhi Zhao, Liju Huang, Xiaoyu Peng, Hailan Chen, Xiaochuan Tang

**Affiliations:** 1 Guangxi Key Laboratory of Animal Breeding, Disease Control and Prevention, College of Animal Science and Technology, Guangxi University, Nanning 530004, China; shulingmeng1115@163.com (S.M.); zhizhao1998@163.com (Z.Z.); huanglg0120@163.com (L.H.); 2 Scientific Research Academy of Guangxi Environmental Protection, Nanning 530022, China; pxyjjvtkb@163.com

**Keywords:** CRISPR/Cas, nucleic acid detection, animal infectious diseases, pre-amplification, read-out method

## Abstract

Increasingly complex epidemics of animal infectious diseases have emerged as a major risk to livestock production and human health. However, current detection methods for animal infectious diseases suffer from shortcomings such as insufficient sensitivity, complicated operation, and reliance on skilled personnel, highlighting the urgent need for novel sensing platforms. CRISPR/Cas systems are adaptive immune systems found in many prokaryotes. Owing to their ability to precisely and reliably target and cleave nucleic acids, the CRISPR/Cas-based nucleic acid detection technology is considered a promising new detection method. When leveraged with a pre-amplification step and established readout methods, CRISPR/Cas-based sensing platforms can achieve a high sensitivity of single-base resolution or attomolar levels on-site. In this review, we first outline the history, working principles, and nucleic acid detection platforms derived from various CRISPR/Cas systems. Next, we evaluate the advantages and limitations of different nucleic acid pre-amplification methods integrated with CRISPR/Cas systems, followed by a discussion of readout methods employed in CRISPR/Cas-based sensing platforms. Additionally, we highlight recent applications of CRISPR/Cas-based sensing platforms in identifying animal infectious diseases. Finally, we address the challenges and prospects of CRISPR/Cas-based sensing platforms for the early and accurate diagnosis of animal infectious diseases.

## 1. Introduction

In 2013, the outbreak of the highly pathogenic avian influenza (HPAI) H7N9 infected a total of 451 people globally and resulted in 165 deaths, with a high case-fatality rate of 36% [1]. In 2018, African swine fever (ASF), caused by the African swine fever virus (ASFV), spread around the globe, causing 99 reported outbreaks in China and more than 800,000 infected hogs [2]. ASF is still one of the most prevalent epidemics in the pig industry without commercial vaccines. In late 2019, there was a worldwide outbreak of the coronavirus disease 2019 (COVID-19), with more than 760 million cases and 6.9 million deaths reported globally as of August 2023 [3], which was later identified by Shi’s team [4] that its sequence identity with a bat coronavirus was as high as 96.2%. Also in 2019, the use of expired disinfectants by workers at the Zhongmu Lanzhou Biological Pharmaceutical Factory led to brucellosis in the air, and aerosols containing brucellosis drifted to the Lanzhou Veterinary Research Institute, ultimately causing brucellosis among 181 students and staffs working there and 3244 residents living around the institute [5]. It is no doubt that animal diseases caused by pathogenic microorganisms not only jeopardize the health of animals and cause huge economic losses but also threaten the lives and health of humans. The accurate and rapid identification of causal pathogens is crucial for the prevention and control of the spread of diseases.

Currently, nucleic acid detection technology is one of the commonly used methods in the diagnosis of animal infectious diseases. The most classic example is the traditional detection technology based on polymerase chain reaction (PCR), including conventional PCR, multiplex PCR, nano-PCR, and fluorescent PCR, etc. Other nucleic acid detection technologies include isothermal amplification technology (IAT), nucleic acid hybridization (NAH), and whole-genome sequencing (WGS), etc. Although PCR is a mature technology and the cost is relatively low, it has certain requirements on the professionalism of the operators and the detection equipment, and it cannot be applied to on-site detection [6]. IAT is free from the reliance on expensive instruments, but it lacks sensitivity [7,8]. NAH has good specificity and high sensitivity, yet requires advanced probes [9,10]. WGS provides accurate results; however, the detection time is long and the cost is high [11,12]. There is still great demand for a simple, rapid, cost-effective, and equipment-independent on-site disease diagnosis technology to accelerate disease management practices while reducing time and labor.

The clustered regularly interspaced short palindromic repeats (CRISPR) and CRISPR-associated proteins (Cas) system can identify the specific nucleic acid sequence of target microorganisms, resulting in the activation of the cis-cleavage and trans-cleavage (also called non-specific cleavage or collateral cleavage) activities which will cleave reporter probe to generate detectable signals. Compared with other detection methods, CRISPR/Cas-based nucleic acid detection technology has the characteristics of rapid detection, independence from special instrument, small reaction volume, good specificity, and high sensitivity and accuracy, demonstrating unique advantages in the diagnosis of animal infectious diseases.

More and more detection sensors are realized by CRISPR/Cas-based nucleic acid detection technologies. For instance, Nouri et al. [13] established the SCAN assay by combining CRISPR/Cas12a with solid-state nanopore sensors. Joung et al. [14] developed the “SHERLOCK testing in one pot-Covid, version2” (STOP Covid.v2) assay platform, which enables one-pot testing using Reverse transcription loop-mediated isothermal amplification (RT-LAMP) of RNA followed by the combination of Cas12b. Ding et al. [15] introduced the all-in-one dual CRISPR/Cas12a (AIOD-CRISPR) one-pot assay platform, which combines recombinase polymerase amplification (RPA) with Cas12a.

The CRISPR/Cas-based nucleic acid detection usually uses Cas9, Cas12, and Cas13 systems, and the detection includes four main processes: nucleic acid amplification, target recognition, nucleic acid cleavage by CRISPR/Cas system, and result interpretation, which is characterized by high sensitivity and high specificity and has been gradually applied in the field of animal infectious disease diagnosis in recent years. This paper first introduces CRISPR/Cas-based nucleic acid detection technology, then summarizes its application in animal infectious diseases, after which it discusses its advantages and limitations in the field of nucleic acid detection, and envisions its future development direction.

## 2. CRISPR/Cas-Based Nucleic Acid Detection

### 2.1. CRISPR/Cas System

The CRISPR/Cas system is a natural immune system found in most archaea and bacteria to defend against invasions by foreign DNA and phages [16,17].

**Figure 1:**
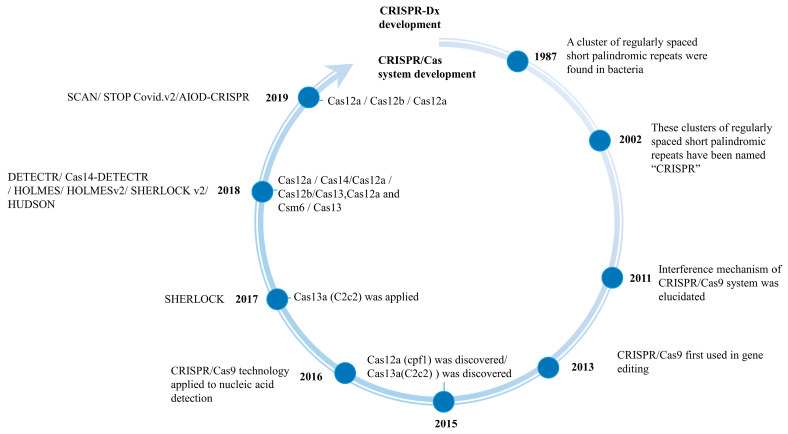
History of CRISPR/Cas-based nucleic acid detection technologies.

The development path of CRISPR/Cas system from the discovery to application in nucleic acid detection is a long process (Figure 1). In 1987, a cluster of regularly spaced short palindromic repeats was discovered in *Escherichia coli* (*E. coli*) [18]. In 2002, in order to understand the structure of their features and to avoid naming confusion, this set of sequences was officially named “CRISPR” by Ruud et al. [19]. In 2011, Emmanuelle Charpentier [20] discovered trans-activating CRISPR RNA (tracrRNA) in the Cas9 system and showed that tracrRNA can base-pair with precursor-CRISPR RNA (pre-crRNA) to form a complex that related to the specific recognition function of CRISPR/Cas9 system. In 2013, Abudayyeh et al. [21] successfully pioneered the adaptation of CRISPR/Cas9 for genome editing in eukaryotic cells, leading to the widespread use of CRISPR/Cas technology in gene editing. It was only in 2016 that Pardee et al. [22] applied CRISPR/Cas9 technology to nucleic acid detection for the first time, detecting zika virus (ZIKV) from the plasma of viremic rhesus monkeys. Since then, the nucleic acid detection function of CRISPR/Cas system has gradually been emphasized. Currently, specific high-sensitivity enzymatic reporter unlocking (SHERLOCK), DNA endonuclease-targeted CRISPR trans reporter (DETECTR), one-hour low-cost multipurpose highly efficient system (HOLMES), and heating unextracted diagnostic samples to obliterate nucleases (HUDSON) are the four representative CRISPR/Cas system-based platforms that apply in nucleic acid detection. SHERLOCK was a nucleic acid detection platform based on CRISPR/Cas13 system that was developed by Gootenberg et al. [23] in 2017, which combines the CRISPR/Cas13a allele-specific sensing capability with a recombinase polymerase amplification (RPA) method to specifically detect DNA and RNA sequences. DETECTR was reported by Chen et al. [24] in 2018, which equips single-strand deoxyribonuclease (ssDNase) and isothermal amplification into the CRISPR/Cas12a system. DETECTR has been used to detect human papilloma virus (HPV) in patient samples with high specificity. In the same year, Harrington et al. [25] used CRISPR/Cas14 to programmatically disrupt DNA and established a novel platform named Cas14-DETECTR, which enables high-fidelity single-nucleotide polymorphism geno-typing. According to the collateral cleavage activity of Cas12a, Li et al. [26] develop the “one-hour low-cost multipurpose highly efficient system” (HOLMES) technology using self-quenched fluorescent ssDNA molecules as probes. Subsequently, Li developed the HOLMESv2 technique using the collateral cleavage activity of Cas12b [27]. Gootenberg et al. [28] reported a multiplex portable nucleic acid detection platform using Cas13, Cas12a, and Csm6 (SHERLOCKv2), which can detect mutations in dengue or ZIKV ssRNA as well as in patients’ liquid biopsy samples by lateral flow. Subsequently, Myhrvold et al. [29] established the HUDSON nucleic acid detection platform by optimizing the previous Cas13-based SHERLOCK platform that simplifies nucleic acid extraction and purification.

**Figure 2:**
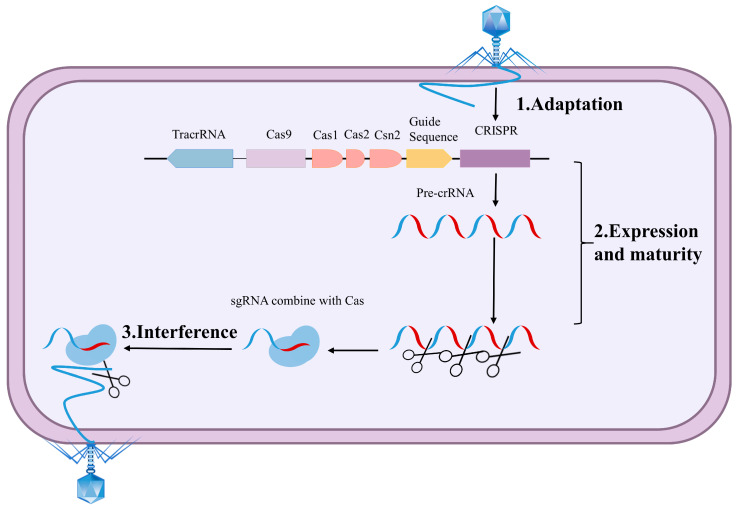
Schematic diagram of the role of the CRISPR/Cas system.

The CRISPR/Cas system primary course of action can be categorized into three stages as shown in Figure 2. The first stage is adaptation. When exogenous DNA or phages invade, the bacterial immune system is activated and Cas protein complex is formed. The Cas protein complex targets the exogenous genome’s proto-spacer sequences [30,31,32,33] and integrates them into the CRISPR sequences [34,35]. The second stage involves crRNA expression and maturation. CRISPR is transcribed into pre-crRNA and pre-crRNA matures under the action of enzymes, resulting in the proto-spacer sequence being transcribed into mature crRNA [36,37,38]. The third stage is the interference target. When bacteria encounter the invasion of homologous exogenous DNA or phages again, the crRNA and tracrRNA undergo local base-pairing to form single-guide RNA (sgRNA). This sgRNA binds to Cas protein to form a complex, and the sgRNA then recognizes the proto-spacer adjacent motif (PAM) sequence, which is immediately adjacent to the original spacer sequence, guiding the Cas protein to localize and bind to the crRNA complementary double-stranded DNA (dsDNA), thereby disabling invading homologous exogenous DNA and phages [39,40,41].

According to the types of Cas effector proteins, the CRISPR/Cas system can be categorized into two main classes and six types [39,40,42] (Table 1). The first class of the CRISPR/Cas system comprises multiple effector proteins that form a complex before the cleavage of the target nucleic acid. The Cas effector proteins in this class include types I, III, and IV, which are represented by Cas3, Cas10, and Csf1, respectively. Each of these proteins performs a distinct function in the effector process. The second major class of CRISPR/Cas systems requires only a single protein to function and includes types II (Cas9), V (Cas12a, Cas12b and Cas14), and VI (Cas13a, Cas13b) [21,40]. In total, 90% of CRISPR/Cas systems belong to the first class, but the second class of CRISPR/Cas systems is mostly applied to CRISPR/Cas-based nucleic acid detection technology because only one Cas effector protein is required and its cleavage efficiency is high [43]. Consequently, the current CRISPR/Cas-based nucleic acid detection platforms are predominantly based on the second class of CRISPR/Cas system, among which Cas9, Cas12, and Cas13 are the most widely used Cas proteins.

### 2.2. Principles of CRISPR/Cas-Based Nucleic Acid Detection

**Figure 3:**
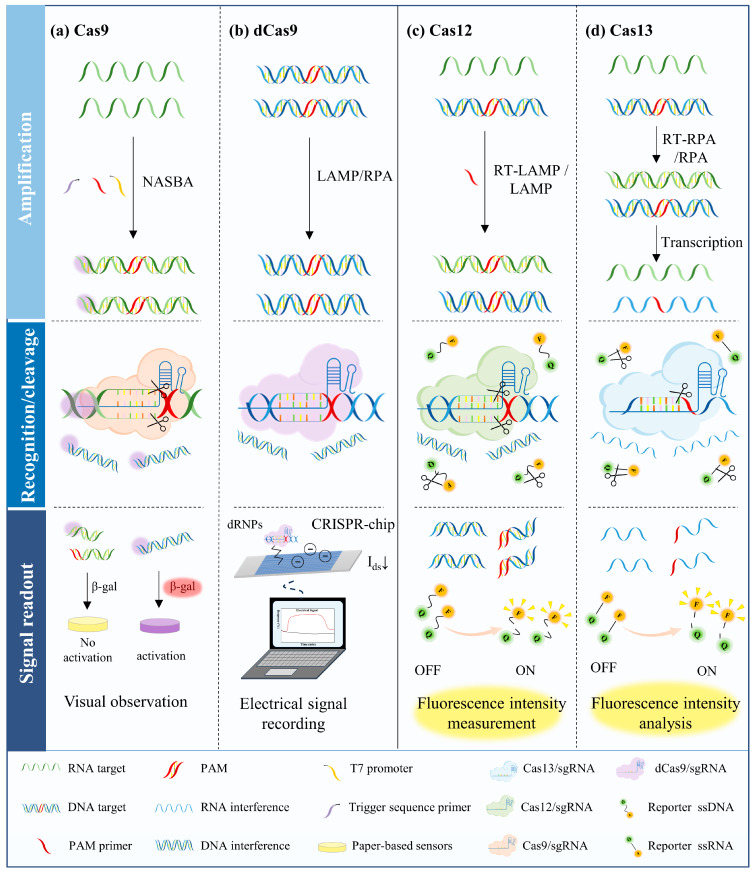
Basic principles of CRISPR-based nucleic acid detection. (**a**) Schematic of the Cas9/sgRNA detection principle. The system integrates isothermal RNA amplification, toehold switch-based RNA sensors, and a paper-based sensor. By leveraging the cis-cleavage activity of Cas9, the results of RNA detection are visualized through color changes on the paper disk. (**b**) Schematic of dCas9/gRNA detection principle. This approach combines isothermal amplification with an electrical signal sensor. Utilizing the targeting capability of dCas9, the detection result is determined by differences in electrical signals. (**c**) Schematic of the Cas12/gRNA detection principle. The collateral cleavage activity of Cas12 is employed to monitor DNA detection results by observing whether the ssDNA signal reporter releases a fluorophore. (**d**) Schematic of the Cas13/gRNA detection principle. The collateral cleavage activity of Cas13 is utilized to detect RNA by determining whether the ssRNA signal reporter is cleaved, thereby generating a fluorescent signal.

According to the collateral cleavage capabilities of the Cas proteins employed, CRISPR/Cas-based nucleic acid detection systems can be categorized into two main types summarized in Figure 3.

The first type is based on the high specificity of Cas9 to recognize and bind dsDNA. CRISPR/Cas9-based nucleic acid detection technologies can be further categorized into two categories; the first utilizes the “cleavage function” of Cas9 and the second leverages the “localization function” of Cas9 or dead Cas9 (dCas9), which possess high specificity for dsDNA recognition and binding [44,45]. Both approaches require the design of specific single-guide RNA (sgRNA) or guide RNA (gRNA) for different targets. When utilizing the “cleavage function”, it mainly relies on DNA endonuclease Cas9; Cas9 binds to and cleaves the target nucleic acids under the guidance of the sgRNA, generating signals that can be detected by certain instruments. For example, the nucleic acid is first amplified by nucleic acid sequence amplification (NASBA), then the trigger sequence is appended to the NASBA-amplified RNA fragments by reverse transcription, and subsequently Cas9 binds and cleaves the target nucleic acid guided by a specific sgRNA, where the truncated sequence is unable to activate the sensor toehold switch, and the color of the paper-based sensor does not change; on the contrary, the non-specific product is not cleaved, and the intact trigger sequence can activate the sensor, resulting in a color change [22] (Figure 3a). On the other hand, when utilizing the “localization function”, it primarily relies on the use of dCas9; dCas9 is unable to cut DNA because it has lost endonuclease activity, but it retains the ability to bind specific DNA sequences. Under the guidance of sgRNA, dCas9 binds to specific DNA sequences, enabling detection through signal conversion mechanisms such as charge modulation, enzyme labeling, fluorescent probes, or chromogenic compounds. The presence or absence of target nucleic acids can then be determined by measuring the resulting signal [46]. For instance, in a graphene field-effect transistor (gFET) detection system, CRISPR/dCas9 is fixed to the chip surface. When dCas9 captures the target DNA, the negative charge of the DNA changes the conductivity of the graphene, thereby outputting a measurable electrical signal [47] (Figure 3b).

The second type is based on the specific recognition and collateral cleavage properties of the Cas12, Cas13, and Cas14 proteins. The difference between the CRISPR/Cas system composed of these proteins and the CRISPR/Cas9 system is that in addition to activating cis-cleavage, non-specific collateral cleavage can also be triggered after recognizing the specific target sequence. Therefore, using the property of trans-cutting, a non-targeted functionalized reporter nucleic acid is added to the reaction system, and when the target nucleic acid is present, the reporter nucleic acid is also destroyed, thus releasing a detection signal. For instance, single-stranded DNA (ssDNA) or single-stranded RNA (ssRNA) labeled with fluorescein and fluorescence quencher at each end is used as a reporter nucleic acid, and the complexes of Cas12, Cas13, and Cas14 proteins with crRNA specifically recognize the amplified target nucleic acid (Cas12 recognizes the DNA, Cas13 recognizes the RNA, and Cas14a recognizes the ssDNA), after which collateral cleavage activity is activated and the reporter nucleic acid is non-specifically cleaved, then the fluorescein and fluorescence quencher are separated, generating a fluorescent signal [48]; therefore, detection is achieved (Figure 3c,d).

### 2.3. Nucleic Acid Amplification for CRISPR/Cas-Based Detection Platforms

**Figure 4:**
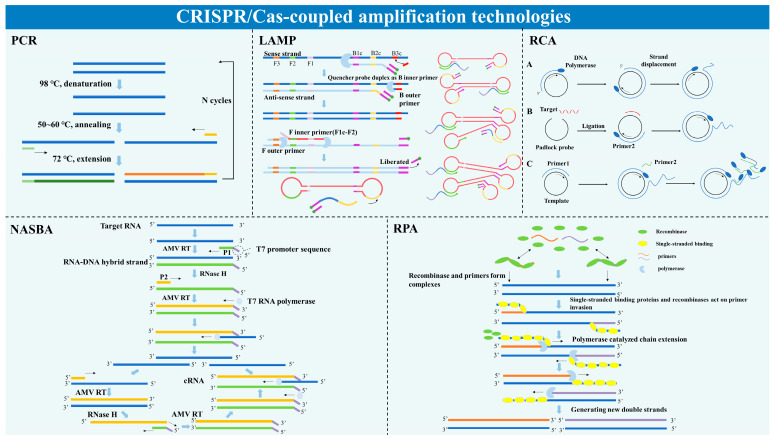
Nucleic acid amplification techniques commonly used in CRISPR/Cas-based nucleic acid detection platforms.

Pre-amplification of target nucleic acids enables CRISPR/Cas-based nucleic acid detection to achieve aM sensitivity and single-base resolution for diagnostic requirements [49]. Therefore, various nucleic acid amplification techniques, such as PCR, NASBA, LAMP, RPA, and RCA, have been frequently equipped with the CRISPR/Cas-based detection process (Figure 4).

#### 2.3.1. PCR

PCR technology has been widely used in nucleic acid amplification since its invention by Saiki [50] in 1988, owing to its high sensitivity and specificity. The principle of PCR is analogous to the natural replication process of DNA, where the DNA double strand is denatured and separated into single strands at a high temperature (95 °C). Then, the single strands are extended into double strands by the addition of designed primers, DNA polymerase, and dNTPs, with the primers binding to the complementary sequences of the DNA single strands at a lower temperature (around 55 °C). At this temperature, the primers anneal to the complementary sequences of the DNA single strands. Finally, the DNA polymerase completes the replication of the DNA strands using dNTPs by the principle of base-complementary pairing at 72 °C. In the context of CRISPR/Cas nucleic acid detection technology, Wang et al. [51] combined CRISPR/Cas9 with PCR and employed PCR for nucleic acid amplification of HPV16 and HPV18 L1 genes in high-risk HPV subtypes. Finally, they utilized CRISPR/Cas9 technology to complete the detection of HPV16 and HPV18 L1 genes with a sensitivity of 0.4 copies/μL, which is almost 200-fold and 10-fold higher than conventional PCR and nano-PCR, respectively. PCR technology, despite being a classic method for nucleic acid amplification, poses significant challenges in terms of instrumentation requirements and operator professionalism, making it unsuitable for widespread application at the grassroots level. Otherwise, the necessity for cycling through multiple temperature steps in PCR typically necessitates a lengthy reaction time of at least 90 min. Consequently, PCR falls short in popularity compared to techniques like LAMP and RPA, which are favored in CRISPR/Cas nucleic acid detection due to their convenience and speed.

#### 2.3.2. NASBA

NASBA is an isothermal nucleic acid amplification technique developed on the basis of PCR. The reaction, which is guided by a pair of primers containing the T7 promoter sequence, is carried out at a constant temperature of 41 °C. The amplification efficiency is higher than that of the conventional PCR method, with strong specificity, high sensitivity, and no special instrumentation required. Pardee et al. [22] first used CRISPR/Cas technology to detect the Zika virus nucleic acid in combination with NASBA, and the detection sensitivity level reaches femtomole (fM) level. Although NASBA is more stable and accurate than traditional PCR technology, the reaction components are more complex, and three enzymes are needed simultaneously, making the cost of the reaction higher. NASBA does not have obvious advantages in the detection of DNA viruses, so NASBA is less frequently used in CRISPR/Cas technology.

#### 2.3.3. LAMP Technology

LAMP technology was invented by Notomi et al. [52] in 2000 as a fast, efficient, sensitive, and specific technique for isothermal amplification of nucleic acids in vitro. LAMP technology requires the design of four specific primers for the six regions of the target genes, and the gene is amplified under the action of strand-displacing DNA polymerase (Bst DNA polymerase) at 60–65 °C. Template, primers, and strand-substituted DNA synthetase can complete the amplification of specific nucleic acids in about 15–60 min. The application of LAMP technology in CRISPR/Cas nucleic acid detection technology is very common. For example, the most classic HOLMES detection platform utilizes LAMP technology for amplifying target nucleic acids [23], and the subsequent HOLMESv2 detection platform combines LAMP amplification of nucleic acids along with bisulfite treatment to accurately quantify the degree of methylation of target nucleic acids [28]. LAMP technology has high amplification efficiency, which can achieve 10^9^–10^10^-fold nucleic acid amplification in about 15–60 min [53], also has the advantages of short reaction time, high specificity, and does not require special equipment. There, it is the nucleic acid amplification technology often used in CRISPR/Cas technology. But at the same time, it has particularly high requirements for primers, which limits its use to some extent.

#### 2.3.4. RPA Technology and RAA Technology

The RPA technology is a nucleic acid isothermal amplification technique developed from the T4 phage DNA replication system [54]. The RPA technique relies on three enzymes: recombinase that binds single-stranded nucleic acids (oligonucleotide primers), ssDNA-binding proteins (SSBs), and strand-displacing DNA polymerases. The reaction takes place at 37–42 °C. The recombinase binds to the primer to form a protein–DNA complex, enabling the primer to search for and locate homologous sequences. After this, a strand-displacement reaction occurs to initiate DNA synthesis, and the displaced DNA strand binds to the SSB, preventing further displacement. The entire reaction process takes 10–40 min. The DETECTR detection platform combines RPA for nucleic acid amplification of the human oncovirus and this platform can achieve aM-level sensitivity [24]. When building the SHERLOCK assay platform, Gootenberg et al. [23] explored combining Cas13a-based assays with different isothermal amplification steps and finally found that RPA provided the greatest sensitivity, enabling sensitivity to the aM level [55,56]. RPA technology has a lower reaction temperature than LAMP and does not require thermal cycling, thus RPA is also commonly used for nucleic acid amplification in CRISPR/Cas-based nucleic acid detection. However, the lower temperature of the reaction also makes the presence of non-specific amplification products unavoidable. Nevertheless, if the specificity of the sgRNA is well-designed, the impact of non-specific amplification products on the CRISPR/Cas reaction can also be mitigated. Subsequently, Recombinase Aided Amplification (RAA) technology was developed based on RPA [56]. Its principle is similar to that of RPA, except that the recombinase used in RAA technology is derived from bacteria or fungi [56], which is less costly compared with RPA technology. In addition, RAA technology uses DNA polymerase, which has a stronger extension rate and strand-replacement ability. At the same time, RAA technology optimizes the ratio of recombinase-SSB, which makes the synergistic effect of recombinase and SSB more efficient [57], such that the amplification time of RAA technology is usually shorter than that of RPA technology [58,59]. There has also been a number of studies applying RAA technology for nucleic acid amplification in CRISPR/Cas-based nucleic acid detection [60,61,62].

#### 2.3.5. RCA Technology

Rolling circle amplification (RCA) technology, a combined amplification and target nucleic acid detection method, was developed by Lizardi et al. [63] to mimic the rolling circle replication process of microbial circular DNA in nature. Using circular DNA as a template, DNA primers are synthesized with strand displacement of the circular DNA template under the action of DNA polymerase with strand-displacement activity to achieve isothermal linear amplification of the circular DNA templates in vitro. Wang et al. [64] used RCA-assisted CRISPR/Cas9 to detect a wide range of extracellular vesicular small RNAs, which can achieve sensitivity at single-base resolution. RCA has high sensitivity and specificity, but the synthesis of probes is expensive, so CRISPR/Cas technologies are also less frequently combined with it.

### 2.4. Signal Readout Patterns of CRISPR/Cas-Based Nucleic Acid Detection Technology

**Figure 5:**
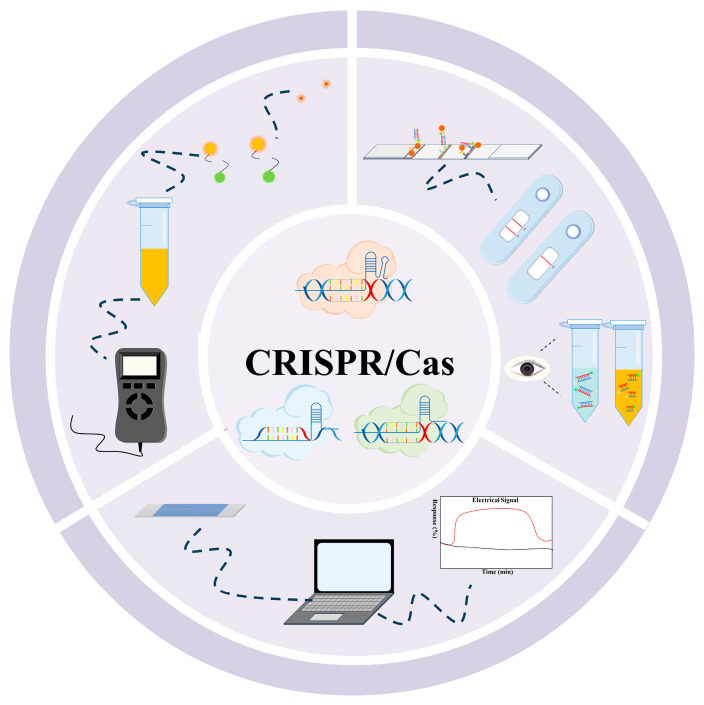
Different signal readout patterns of CRISPR/Cas-based nucleic acid detection technology.

The recognizing and cleavage results of target nucleic acids are not readable and so are required to be transferred to observable or detectable signals, such as colorimetric signal, fluorescence signal, and electrical signal (Figure 5).

#### 2.4.1. Colorimetric Signals

Colorimetric signals can either be observed directly by the naked eye or with the aid of a photoelectric colorimeter. In CRISPR/Cas-based nucleic acid assays, the colorimetric signals are often presented in combination with a Lateral flow assay (LFA) [65]. This is achieved by adding fluorescein (FAM) and biotin-labeled DNA or RNA (FAM-ssDNA-Biotin/FAM-ssRNA-Biotin) as reporter probes to the reaction system. Gold nanoparticles (AuNPs) labeled with anti-fluorescein antibody are used as a binding pad for the test strip, while streptavidin, which can bind to biotin, is encapsulated on the quality control line. The secondary antibody is encapsulated on the detection line. The Cas12 or Cas13 system non-specifically cleaves the reporter gene, and the results are interpreted by observing the change in the color of the detection line on the test strips after the reaction solution has been chromatographed [66,67,68,69]. Lu [67] developed a test strip for rapid detection of African swine fever virus based on Cas12a using AuNPs labeled with anti-FITC antibody. The sensitivity of the test strip was 200 copies/sample, which was comparable to that of RT-PCR. There are also those who do not observe the results through test strips but directly observe the color change in the reaction solution in the centrifuge tube to read the results. For example, Yuan et al. [70] designed an AuNPs-DNA probe that can undergo changes in aggregation behavior after trans-cleavage, which enables naked-eye gene detection (using transgenic rice, ASFV and miRNAs as models) to be completed within 1 h.

#### 2.4.2. Fluorescence Signals

Fluorescence signals are the classical mode of expression of CRISPR/Cas nucleic acid assay results. A fluorescent probe is used in the reaction system, with one end of the probe labeled with a fluorescent reporter group and the other end labeled with a fluorescence quenching group. When the probe is intact, the fluorescence signal emitted by the reporter group is absorbed by the quenching group, resulting in no fluorescence signal being generated. When the Cas12 or Cas13 system is used, the fluorescent probe can be destroyed by non-specific cleavage, causing fluorescent signals to be generated. These signals are then detected by a fluorescence detector to achieve qualitative and quantitative detection [71]. Wang et al. [72] created a diagnostic technique based on the RPA-CRISPR/Cas system to identify the Nc5 gene of *Neospora caninum* (*N. caninum*). The technique uses a fluorescent reporter system and a LFA biosensor to display results. When using a fluorescent reporter system, the limit of detection (LOD) was as low as one parasite per milliliter, a 10-fold improvement over the LOD using the LFA biosensor.

#### 2.4.3. Electrical Signals

The electrical signal primarily converts the DNA or RNA signal in the CRISPR/Cas reaction into an electrical signal, and the strength of this electrical signal is detected to determine the concentration level of the target. There are various sensors for electrical signals, including electrochemical sensors [73,74], nanopore sensors [75], graphene field-effect transistor (GFET)-based sensors [75] and conductivity sensors combined with DNA gels [76]. He et al. [74] modified the surface of gold electrodes with methylene blue (MB, 3′-terminal) and thiol groups (5′-terminal)-labeled hairpin DNA (hpDNA) probe, and utilized the non-specific cleavage activity of the Cas12a system to cut the hpDNA. This led to the dissociation of MB from the hpDNA on the electrode surface, resulting in a sharp decrease in the electrochemical signals. Finally, the results were read by detecting the change in these electrochemical signals.

#### 2.4.4. Multi-Signal Mode Outputs

Most general CRISPR/Cas nucleic acid detection platforms employ a signal reading mode, but there are also multi-signal mode CRISPR/Cas detection methods. Multi-signal mode outputs combine multiple signals to make up for the shortcomings of a single signal output by complementing the data, thus improving the convenience and sensitivity of detection. Zhao et al. [77] developed a biosensor based on CRISPR/Cas12a with dual-mode output, combining fluorescent and colorimetric signals, which can detect free DNA (cfDNA). The biosensor can be achieved through the colorimetric signal first direct observation of the results, and through the fluorescent signal to improve the detection sensitivity.

### 2.5. Matching Strategies for CRISPR/Cas-Based Diagnostic Technology

As described in Section 2.2, Section 2.3 and Section 2.4, the performance of CRISPR/Cas-based diagnostic technologies depends on the synergistic interaction of three factors: Cas protein characteristics, nucleic acid amplification efficiency, and signal output sensitivity. Although the matching of these three factors is not strictly required, certain optimal combinations do exist in practice.

For natural Cas9 proteins, DNA target detection typically employs a strategy that combines RPA or PCR with T7 in vitro transcription to generate RNA-DNA hybrid chains containing PAM sites. RNA virus detection, meanwhile, can be achieved through RT-RPA or NASBA, which involves the introduction of specific trigger sequences. Additionally, the cis-cleavage characteristic of natural Cas9 proteins primarily acts on the target itself. The cleaved nucleic acid fragments remain in situ, enabling hybridization staining and signal enrichment. Therefore, colorimetric output (e.g., lateral flow test strips) is ideal for CRISPR/Cas9 detection systems.

CRISPR/Cas12 and CRISPR/Cas13 utilize their trans-cleavage activity to introduce specific reporter molecules into the detection system to generate a signal. The Cas12 system specifically recognizes DNA targets, generating signals through the cleavage of ssDNA, forming a golden combination with LAMP and fluorescence detection. Cas13 specifically recognizes RNA and its trans-cleavage of ssRNA is naturally compatible with RPA technology. The signal amplification effect produced by combining the two makes fluorescence detection the preferred option. Cas12 and Cas13 can also, of course, be combined with colorimetric and electrical signal outputs. For example, the reaction temperature of RPA is similar to the incubation temperature of electrochemical electrodes. Although colorimetric signals are less sensitive, they are better suited to on-site detection.

Although there is a certain degree of compatibility between Cas proteins, nucleic acid amplification methods, and signal outputs, this relationship is not fixed. In practical applications, adjustments must be made flexibly based on different detection requirements (such as sensitivity, speed, cost, application scenarios, etc.).

## 3. Application in the Diagnosis of Animal Infectious Diseases

With the development of the breeding industry, the prevention and control of animal infectious diseases have been increasingly emphasized. However, due to the high variability of viruses, the lack of effective vaccines, and the absence of high medical standards, animal diseases are becoming increasingly complex which increase the difficulty in disease prevention and control. Consequently, the rapid and accurate identification of the causal pathogens is critical for the development of preventive and control measures. Unfortunately, for the healthy growth and reproduction of livestock and poultry, most farms are established in remote areas, which makes some of the traditional diagnostic methods unable to meet the needs of timely and rapid clinical disease diagnosis. CRISPR/Cas-based nucleic acid detection is fast, simple, accurate, sensitive, and low-cost, making it highly suitable for the timely analysis of viruses, bacteria, parasites, and other pathogen infections. Table 2 summarizes the application of CRISPR/Cas nucleic acid-based assays in diagnosing animal infectious diseases in recent years.

### 3.1. Diagnosis of Bacterial Epidemics

Bacterial epidemics are one of the common diseases endangering animal health, and they are particularly prevalent in developing countries. Common bacterial pathogens in animals include *Salmonella* [102], *E. coli* [103,104], and *Staphylococcus aureus* (*S. aureus*) [105,106], etc. Their infection can cause gastroenteritis, mastitis, arthritis, and other diseases in livestock and humans. Traditional diagnostic methods and serum immunoassay are mostly used to diagnose bacterial diseases, but both of them are time-consuming and cumbersome. As an emerging technology, CRISPR/Cas-based nucleic acid detection technology is gradually being applied in bacterial detection due to its advantage of rapid detection.

**Figure 6:**
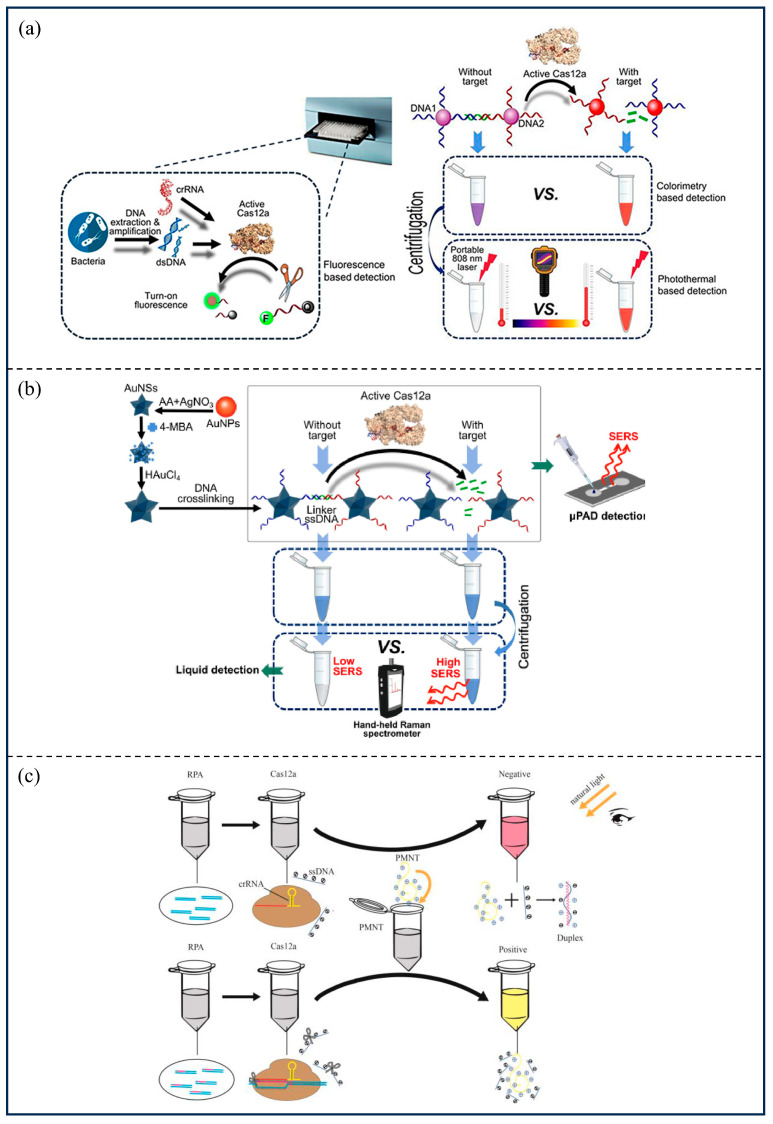
Application of CRISPR/Cas to bacterial infectious diseases in animals. (**a**) The inserted figures are reprinted with permission from Ref. [79]. Copyright 2021, ACS; (**b**) Reprinted with permission from Ref. [80]. Copyright 2022, Elsevier; (**c**) Reprinted with permission from Ref. [81]. Copyright 2023, MDPI.

Zhang et al. [78] utilized dCas9’s recognition of sequences to accurately detect *Mycobacterium* tuberculosis DNA. By using a pair of dCas9 proteins linked to luciferase, luciferase induces luminescence when dCas9 locates a target sequence defined by the sgRNA. The sensitivity of the assay could be as high as 1 copy/tube. Ma et al. [79] developed a CRISPR/Cas12a-driven dual-mode biosensor that can be used to detect *Salmonella*. Specifically, after *Salmonella* nucleic acid were amplified, the specific invA sequence triggered CRISPR/Cas12a to trans-cleavage ssDNA that connected two (AuNPs). The color change could be observed with the naked eye or recorded using a portable colorimeter (Figure 6a). For these two detection modes, the detection limits and linear dynamic detection ranges were 1 CFU/mL and 10^0^–10^8^ CFU/mL, respectively. Zhuang et al. [80] combined CRISPR/Cas12a with surface-enhanced Raman scattering (SERS) to design the RPA-integrated microfluidic paper-based assay device (μPAD). The SERS nanoprobe is attached to ssDNA, when the target is present, Cas12a cleaves ssDNA, releasing the SERS signal. Finally, the concentration of the target is determined by μPAD and monitored by a Raman spectrometer (Figure 6b), allowing for the detection of *Salmonella typhi* in 45 min, with a detection limit of approximately 3–4 CFU/mL and a linear dynamic detection range of 1–10^8^ CFU/mL. Jiang et al. [81] utilized CRISPR/Cas12a-poly[3-(3′-N,N,N-triethylamino-1′-propoxy)-4-methyl-2,5-thiophene hydrochloride] (PMNT) for the rapid visual detection of *E. coli* O157:H7 after mixing the CRISPR/Cas12a system with ssDNA ligated to PMNT. Based on PMNT conformational modification of the conjugated backbone, the solution eventually shows a yellow color in the absence of the target DNA, enabling colorimetric DNA detection (Figure 6c). Xu et al. [82] investigated a rapid detection method for pathogenic *Bacillus anthracis* in 2023, based on the DETECTR nucleic acid detection platform, with a sensitivity approaching 2 copies. The study by Xu et al. [83] shows that the RPA-CRISPR/Cas12a diagnostic method, developed using fluorescence and electrical signal readout, was able to rapidly and accurately detect four *Brucella* species in blood and milk samples. Compared to real-time fluorescent quantitative PCR (qPCR), this technique utilized a dual-signal readout approach to improve the accuracy of the assay, with sensitivities of up to 2 copies/reaction of positive reference plasmid in both modes.

CRISPR/Cas technology has numerous applications in the diagnosis of bacterial infectious diseases in animals, primarily based on Cas12 system, with colorimetric or fluorescent signal output. It offers shorter detection time, simple operation, and sensitivity comparable to or even exceeding that of PCR.

### 3.2. Diagnosis of Viral Epidemics

Viral infectious diseases in animals are characterized by rapid transmission, widespread prevalence, high infectivity, high mortality, and difficulty in prevention and treatment. Foot-and-mouth disease [107], Influenza [108,109], and ASF [110] are common viral infections, while some infectious diseases, such as HPAI, are characterized by high viral variability and extremely rapid transmission, which can lead to large-scale or even nationwide or worldwide pandemic in a short time. The current methods for diagnosing viral infectious diseases in animals mainly include morphological examination of viruses, isolation and culture of viruses, serological methods, and molecular diagnostic techniques. However, morphological examination of viruses is still difficult; not only are the isolation and culture of viruses time-consuming, but also factors such as improper handling of samples, poor staining, and manipulation errors during observation can affect the results. While serological methods are more sensitive, they are more cumbersome to operate. Molecular diagnostic techniques, such as PCR, are highly sensitive but require specialized instrumentation and trained personnel. Therefore, in the diagnosis of animal viral infectious diseases, there is an urgent demand for rapid, highly sensitive detection methods to enable timely diagnosis. CRISPR/Cas-based nucleic acid detection technology has the potential to overcome the shortcomings of these existing tests.

**Figure 7:**
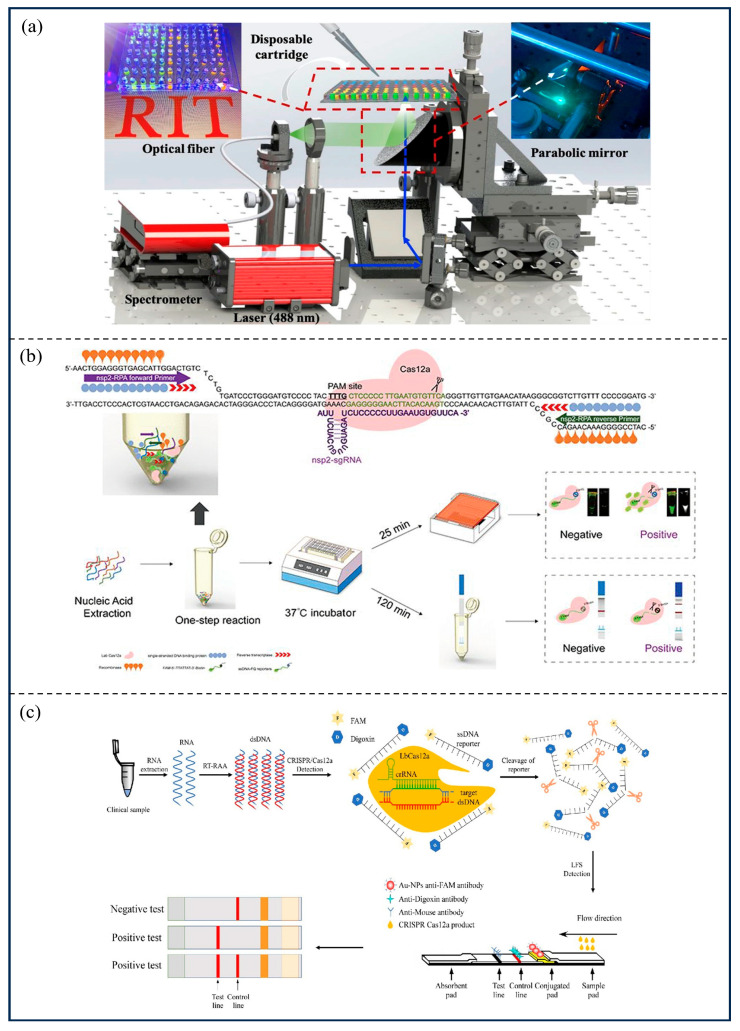
Application of CRISPR/Cas to bacterial infectious diseases in animals. (**a**) The inserted figures are reprinted with permission from Ref. [84]. Copyright 2020, Elsevier; (**b**) Reprinted with permission from Ref. [86]. Copyright 2021, Elsevier; (**c**) Reprinted with permission from Ref. [88]. Copyright 2022, Frontiers.

For viral epidemics, the earliest application of CRISPR technology for the detection of viral nucleic acids was the paper-based sensor developed by Pardee et al. [22] in 2016, based on CRISPR/Cas9 system, for the rapid detection of ZIKV. After the RNA of ZIKV was amplified by nucleic acid sequence-based amplification (NABSA), the CRISPR/Cas9 system was applied to cleave the viral nucleic acid, followed by detection using a paper-based sensor. The sensor can discriminate between viral strains with single-base resolution. The SHERLOCK platform, based on Cas13, was established by Myhrvold et al. [29], which can detect ZIKV and dengue virus (DENV) at concentrations as low as 1 copy/μL. Leveraging the property of the CRISPR/Cas12a system to cut ssDNA in trans, He et al. [84] developed a high throughput and full solution-phase ASFV detection system (Figure 7a). The results of the assay were read by a fluorescent reporter molecule and a detection limit of 1 pM was achieved in 2 h in the absence of nucleic acid amplification. Additionally, the ternary Cas12a/crRNA/ASFV DNA complex is highly stable at 37 °C and continues to cleave the ssDNA reporter gene even after 24 h of incubation, allowing the detection limit to be increased to 100 fM. Lu et al. [67] combined RPA technology with LFA to investigate a portable paper-based diagnostic technology based on Cas12a for rapid detection of ASFV with sensitivity as low as 200 copies/reaction, which is comparable to that detected using qPCR methods. For porcine reproductive and respiratory syndrome (PRRS), CRISPR/Cas13a-based [85] or CRISPR/Cas12a-based [86] diagnostics have been established. The CRISPR/Cas13a-based PRRSV assay has a sensitivity of 172 copies/reaction, which is comparable to reverse transcription-qPCR (RT-qPCR), while the CRISPR/Cas12a-based assay has a much higher sensitivity, achieving single copy within 25 min (Figure 7b). For porcine epidemic diarrhea virus (PEDV), a reverse transcription-enzymatic recombinase amplification (RT-ERA)-CRISPR/Cas12a detection system [87] technique with a detection limit of 2 copies/reaction was developed. This can be used to differentiate between attenuated PEDV vaccine strains and wild-type virus strains. A reverse transcription-Recombinase Aided Amplification (RT-RAA)-CRISPR/Cas12a platform was established for the S gene to detect GII PEDV so as to determine whether pigs should be immunized with the CV777 vaccine [88]. The platform, when combined with LFA, was read with AuNPs-labeled anti-FAM antibodies with a detection limit of 1 × 10^2^ copies/reaction (Figure 7c). Liu et al. [89] developed a single-tube multiplex RT-LAMP-Cas12a diagnostic that simultaneously detects PEDV, transmissible gastroenteritis virus (TGEV), porcine deltacorona virus (PDCoV), and swine acute diarrhea syndrome coronavirus (SADS-CoV), with a detection limit of 1 copy/reaction and a detection time of 83 min. Zhang et al. [90] optimized the CRISPR/Cas12a system and combined it with ERA to diagnose porcine circovirus type 3 (PCV3) with an assay that could detect PCV3 in genomic DNA containing at least 7 copies in less than 1 h. In 2022, Li et al. [91] developed a method for the detection of Crimean–Congo hemorrhagic fever virus (CCHFV) using Cas13a-based SHERLOCK technology, which detects 1 copy/μL of viral RNA in 30–40 min. Xu et al. [92] developed a single-tube rapid visualization assay based on RT-LAMP and CRISPR/Cas12a targeting, which successfully detects the C-gene sequence of Japanese encephalitis virus (JEV) RNA with 8.97 or more copies in approximately 60 min in the field. Fluorescence intensity measurements and direct visualization with the naked eye were consistent with the results of qPCR analysis. Wei et al. [93] developed an easy-to-handle palm-sized pouch called the At-Home Self-Testing Monkeypox (MPXV) and Instant Care Pouch (MASTR Pouch). In this MASTR Pouch, rapid and accurate visual detection of MPXV was achieved by combining RPA with the CRISPR/Cas12a system. From virus particles lysis to naked eye reading, the MASTR Pouch completes the analytical process in just four simple steps within 35 min and can detect 53 Mpox pseudovirus particles (10.6 particles/μL) in exudate.

Viral infectious diseases are extremely harmful to both animals and humans, and CRISPR/Cas-based nucleic acid detection technologies have been widely used in the diagnosis of these diseases, primarily targeting highly contagious common viruses with high sensitivity and good specificity. Furthermore, some CRISPR/Cas technologies even enable the simultaneous detection of up to three viruses, which can be a significant advantage in the detection of animal viruses.

### 3.3. Diagnosis of Parasitic Epidemics

Parasitic diseases are conditions in which the invasion of parasites leads to pathological changes and clinical symptoms. Their epidemiological characteristics encompass endemicity, seasonality, and natural origin. Common parasitic diseases include, among others, *Ascariasis* [111], *Schistosomiasis* [112], and *Hepatic schistosomiasis* [113] and these infections can cause inflammation or organ damage. Current methods of parasite diagnosis involve blood smears, urine samples, or fecal examinations, which, although relatively simple to perform, typically necessitate microscopic observation, rendering them less suitable for on-site detection. Additionally, CRISPR/Cas-based nucleic acid diagnostics have been explored as a potential diagnostic tool for parasitic diseases.

**Figure 8:**
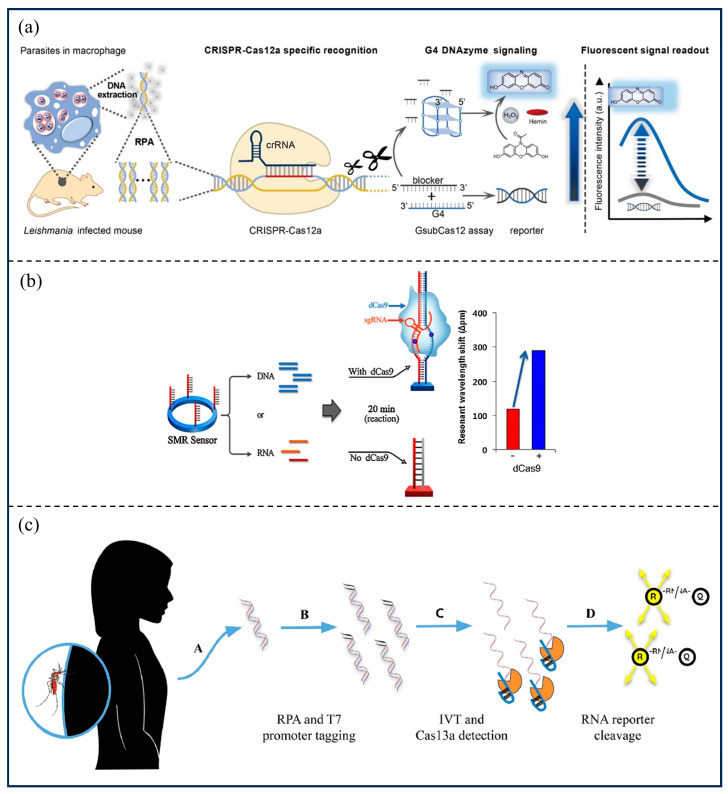
CRISPR/Cas technology in parasite detection. (**a**) The inserted figures are reprinted with permission from Ref. [94]. Copyright 2022, ACS; (**b**) Reprinted with permission from Ref. [95]. Copyright 2018, Elsevier; (**c**) Reprinted with permission from Ref. [97]. Copyright 2021, Elsevier.

Gao et al. [94] utilized G-quadruplex deoxyribonuclease (G4 DNAzyme) as a reporter gene for CRISPR/Cas12 to diagnose unicellular parasitic infections. Compared with an assay using a fluorescent reporter gene, the sensitivity of detecting *Leishmania donovani* (*L. donovani*) when G4 DNAzyme was used as a reporter gene increased by 5-fold (Figure 8a). Koo et al. [95] detected tick-borne diseases, including Scrub typhus (ST) and severe fever with thrombocytopenia syndrome (SFTS), based on a CRISPR/dCas9-mediated biosensor. The single-molecule sensitivities for the detection of ST and SFTS are, respectively, 0.54 aM and 0.63 aM, which is 100-fold more sensitive than qPCR detection (Figure 8b). Cherkaoui et al. [96] developed a diagnostic test, CATSH, for portable real-time fluorescence detection of *Schistosoma haematobium* (*S. haematobium*) using RPA, combined with the CRISPR/Cas12a system, and obtained results in less than 2 h with the minimum amount of gDNA detected being 10 fg/reaction. For *Plasmodium*, Cunningham et al. [97] developed a malaria assay combining RPA, CRISPR-RNA base-pairing and Cas13a cleavage activity based on the SHERLOCK principle, which can be used to detect all known *Plasmodium* species that cause malaria in humans (Figure 8c). Similarly, Lee et al. [98] utilized the SHERLOCK platform for the detection and differentiation of a wide range of *Plasmodium* species, including *Plasmodium falciparum* (*Pf*), *Plasmodium vivax* (*Pv*), and *Plasmodium ovale* (*Po*), which are capable of detecting less than 2 parasites/μL of blood. For *Toxoplasma gondii* (*To*), corresponding detection techniques based on CRISPR/Cas12a or CRISPR/Cas13a systems were also developed [99,100,114], which meet the requirements for rapid and sensitive detection in the field. In addition, Kanitchinda et al. [101] established a fluorescent assay platform combining RPA and CRISPR-Cas12a to detect *Enterocytozoon hepatopenaei* (*EHP*) in shrimp, which can detect as few as 50 copies of DNA.

CRISPR/Cas-based nucleic acid tests have been used less frequently for the detection of parasitic diseases, but their sensitivity is superior to that of current parasite detection methods, and they enable timely detection in the field, which is not available with current parasite detection methods.

## 4. Challenges and Prospects

In the aspect of animal infectious disease detection, traditional pathogenicity detection remains the gold standard for pathogen detection. However, the traditional detection method is time-consuming and primarily suitable for easily identifiable pathogenic microorganisms, as it often necessitates culturing the pathogens over time and subsequently making judgments based on the observation of their morphology. Additionally, the traditional detection process has high requirements and does not apply to the timely diagnosis of clinical diseases. Currently, the detections of animal infectious diseases primarily rely on serologic and modern molecular biology methods. Compared with the traditional pathogenetic diagnosis, serological diagnosis primarily based on the specific reaction between antigens and antibodies takes significantly less time. Some serological methods, such as hemagglutination, hemagglutination inhibition test, ELISA, and immunochromatography, do not rely on specialized or expensive instruments, and exhibit better specificity and sensitivity. Nevertheless, due to the potential cross-reaction of antigens and antibodies, false-positive results can occur. Modern molecular biology testing primarily involves nucleic acid detection, but traditional PCR operations are cumbersome, time-consuming, and costly. Furthermore, traditional PCR relies heavily on specialized equipment and trained personnel. Simplifying the nucleic acid amplification steps to eliminate the reliance on expensive instruments is crucial, as isothermal amplification technology alone is insufficient in terms of sensitivity and specificity in traditional PCR. While gene sequencing offers high accuracy, it is also time-consuming and expensive. In contrast, CRISPR/Cas-based nucleic acid detection technology relies on the specific recognition of Cas protein, ensuring the specificity of detection. Its sensitivity allows for the detection of pathogens at the single-copy level or aM level and most detections are completed within 15 to 60 min, making it both highly sensitive and rapid. This technology can be performed outside of a professional laboratory environment and does not require costly instruments or specialized personnel, making it highly convenient. Furthermore, CRISPR/Cas-based diagnostic technologies have demonstrated a high level of technological maturity due to their well-defined detection principles, simple operational systems, and easily accessible raw materials. In recent years, this technology has been accelerating its transition from laboratory research to clinical applications. As of 2025, multiple platforms including SHERLOCK™ (Cas13-based), DETECTR™ (Cas12-based), and HOLMES™ have received emergency use authorization from the US FDA or China’s NMPA, with several CRISPR nucleic acid detection kits now commercially available. Compared to standard PCR testing, CRISPR diagnostics demonstrate significant cost advantages. Research indicates that the DETECTR system developed by Mammoth Biosciences can achieve a per-test cost of approximately USD 1 under scaled production conditions, substantially lower than conventional qPCR testing [28]. Therefore, when comparing CRISPR-based nucleic acid detection technology with other methods, it exhibits clear advantages in specificity, sensitivity, detection time, cost, and the absence of the need for expensive instruments and specialized personnel (Table 3).

Although CRISPR/Cas-based nucleic acid detection technologies have the advantages of being rapid, highly sensitive, highly specific, requiring no special instrumentation, and low cost, they also have some limitations, including optimization of the one-pot reaction, sample pre-treatment problems, off-target effects, and sequence limitations. Furthermore, they are still not able to provide high-throughput composite assays [115,116,117,118].

### 4.1. Optimization of the One-Pot Reaction and High-Throughput Detection

Since CRISPR/Cas-based nucleic acid detection technology usually requires a pre-amplification step to increase the concentration of target nucleic acids, this makes it difficult for the non-specific cleavage of CRISPR/Cas system to be carried out in one pot; this not only increases the chances of aerosol contamination and prolongs the detection time, but also makes CRISPR/Cas-based nucleic acid detection technology incapable of high-throughput compound detection [119].

The one-pot reaction based on CRISPR/Cas nucleic acid detection technology simplifies the separate two-step reaction process to be performed in the same system. By realizing the amplification of target nucleic acids, activation of the CRISPR/Cas system, and signal generation in the same reaction system, the efficiency and sensitivity of the experiments have been greatly improved, while sample handling, operation steps, and risk of contamination have been reduced, which in turn facilitates rapid and accurate molecular detection, and also provides the possibility of high-throughput detection. There have also been a number of studies based on isothermal nucleic acid amplification technology with CRISPR/Cas systems to realize one-pot reactions, such as the aforementioned STOP Covid.v2 [14] and AIOD-CRISPR [15]. However, CRISPR/Cas one-pot diagnostics, despite being more straightforward than the two-step method, have been observed to exhibit reduced sensitivity and lengthier detection times [120]. Yin Hao’s team dissected the factors affecting the diagnostic sensitivity and detection time of the one-pot assay and found that both Cas12b and Cas13a impaired the amplification of the target sequence. In the one-pot assay based on Cas12b, isothermal amplification and Cas protein cleavage occur simultaneously. The high affinity of the classical PAM sequence to Cas protein makes for high activity of Cas protein cleavage in CRISPR detection, and the substrate cleavage speed is faster than the substrate amplification speed, such that the amplified substrate will be consumed rapidly. Cas13a also directly degrades viral RNA and interferes with sequence amplification [120,121]. Therefore, the CRISPR/Cas one-pot reaction needs to be further optimized to achieve rapid and highly sensitive nucleic acid detection.

In terms of optimizing the reaction system of the one-pot method, Yin Hao’s research team found that when using a suboptimal PAM sequence with lower affinity to Cas12b protein (adjusted the bases in the classical PAM), the cleavage activity of Cas proteins in the CRISPR assay could be significantly reduced, thus ensuring that there is enough amplification substrate for a thermostable amplification reaction, which in turn improves the assay’s sensitivity and reaction speed. They ensured that the RNA transcribed from the DNA amplicon was reverse-complementary to the viral RNA by incorporating a reverse primer for the T7 promoter sequence for amplification, preventing the cleavage of the RNA by Cas13a. Moreover, studies have shown that increasing the solution viscosity or density through the addition of additives (e.g., glycerol [122], sucrose [123], or agarose [124]) enables phase separation in one-pot CRISPR assays, thereby alleviating the CRISPR system’s inhibitory effect on nucleic acid amplification. Hu et al. [125] designed a nucleic acid strand with a photocleavable (PC) connector that almost temporarily silences CRISPR-Cas12a activity. When the amplification product is sufficient, 365 nm UV light is used to reactivate Cas12a, avoiding the interference of Cas12a with the nucleic acid amplification process. In addition to optimizing the reaction system, other researchers have proposed one-pot reactions through amplification-free detection platforms [121]. The establishment of amplification-free CRISPR/Cas detection platforms is mainly achieved through several strategies, such as optimizing crRNA and signal-reporter molecules for CRISPR/Cas reactions, digitizing sensitive detection platforms, coupling sensitive signal sensors, and designing cascade reactions to achieve output signal amplification [126]. A number of amplification-free CRISPR/Cas nucleic acid detection technologies based on the above strategies have been investigated [126,127]. For instance, Shinoda [128] combined CRISPR/Cas13 RNA detection with microcompartment array technology to create a CRISPR-based amplification-free digital RNA detection platform “SATORI”; Sun [129] achieved a one-pot reaction by designing an LNA-modified split activator (CALSA) as a cascade signal amplification tool to drive CRISPR/Cas autocatalytic amplification. Other researchers have employed magnetic enrichment [130,131] or other techniques to concentrate targets, simplifying sample pre-treatment steps while eliminating the need for nucleic acid amplification.

Microfluidics is a technology that allows for precise manipulation of fluids at the micron and nanometer scale, enabling microfabrication on chips measuring a few square centimeters. This technology has applications in a range of fields, including sample preparation, reactions, separations, and assays. Researchers have discovered that combining microfluidics with the CRISPR system to transfer the nucleic acid analysis process to the chip allows for rapid, high-throughput, integrated, multiplexed, and digital CRISPR/Cas-based nucleic acid detection. This solution addresses the limitation of CRISPR/Cas-based nucleic acid detection technology in achieving high-throughput composite detection [69,132,133]. Currently, some researchers have combined microfluidics with Cas13-based assays, or integrated microwell arrays with Cas13-based assays, to achieve high-throughput composite detection of specific pathogens [134,135,136].

### 4.2. Off-Target Effects and Sequence Limitations

On the one hand, the specificity of the CRISPR/Cas system depends on the precise sequence recognition by sgRNA. However, when the designed sgRNA forms mismatches with non-targeting sequences, it results in Cas cutting sequence at the wrong site or modifying the non-target genes as well as leading to activation of trans-cleavage failures, which is the off-target effect of the CRISPR system. The off-target effect is an inherent defect of the CRISPR system, due to the high tolerance of Cas proteins to off-target structures (especially Cas9 [137]), which allows Cas proteins to undergo cleavage when the distal region of the PAM of the sgRNA targeting sequence is mismatched even if the mismatch is as high as 3–5 bp [138]. The off-target effect usually leads to a decrease in the specificity of the CRISPR/Cas system and a decrease in the accuracy of the results. On the other hand, the target recognition requirement of PAM for Cas creates sequence constraints, such as the required PAM sequence for Cas9 is NGG (N for A, T, C, and G), but this specific PAM sequence is sometimes difficult to achieve in single-nucleotide polymorphism (SNP) or short sequence assays, which limits the range of target sequences that can be recognized by the CRISPR/Cas system. However, in the diagnosis of animal infectious diseases, the ability to detect single-base resolution is important in improving diagnostic accuracy, monitoring pathogen variation, distinguishing between vaccine and wild viruses, and enabling individualized treatment.

The off-target effect is associated with the designed sgRNA, PAM, and Cas nuclease [139,140,141,142]. Researchers may ameliorate the problem of off-target effects by carefully designing sgRNAs to improve specificity [143], modulating Cas–sgRNA complex concentrations, modifying Cas enzymes, and using off-target prediction platforms such as CRISPR-DIPOFF [144], CrisprSQL [145], and qEva-CRISPR [146]. Alternatively, in order to solve the problem of sequence limitation, researchers have proposed some strategies regarding the establishment of sequence-dependent (PAM-free) one-step CRISPR nucleic acid detection systems. These strategies mainly involve the introduction of PAM sequences in the design of amplification primers; for example, the PAM sequences required for Cas protein recognition are designed into the amplification primer sequences [147] or into the 5′ end sequences of Flap primers [148], which bypasses the dependence of Cas proteins on PAM sequences.

### 4.3. Sample Pre-Treatment Problems

Sample pre-treatment is crucial for the step of nucleic acid amplification, as it separates the components to be tested from the matrix in complex samples, improves nucleic acid extraction by purifying and enriching the target, and ultimately affects the results of the assay. Current sample pre-treatment methods include grinding, ultrasonication, repeated freezing and thawing, chemical, and enzymatic methods, most of which are cumbersome to operate. Moreover, in many CRISPR/Cas-based nucleic acid detection technologies, the sample pre-treatment and detection processes are completed in two separate steps, increasing the risk of contamination. One-pot assays combine the sample pre-treatment and detection processes, and in some methods samples can be detected without special pre-treatment. However, due to the absence of nucleic acid amplification, these assays tend to have lower detection sensitivity [149]. The aforementioned magnetic bead enrichment of samples instead of nucleic acid amplification reduces inhibitor interference while achieving targeted enrichment and also improves the sensitivity and specificity of the one-pot assay [130,131]. However, all of these methods are specific to a particular sample type and are not universally applicable in the face of diverse samples. Therefore, simplifying the sample pre-treatment steps, developing applicable processing techniques, and even realizing the direct detection of samples are still the directions that need to be investigated in CRISPR/Cas-based nucleic acid detection technology.

### 4.4. Other

In addition, it is worth mentioning that last year, Liu Liang’s team [150] proved that cas9 actually has para-cropping activity, and found that the para-cropping activity is activated by target ssDNA, dsDNA, and ssRNA, but can only efficiently trans-cleave T- or C-rich ssDNA substrates, and that the trans-cropping of cas9 guided by the primitive crRNA and tracrRNA, rather than the chimeric sgRNA, has a better catalytic effect. At the same time, the team developed nucleic acid detection methods DACD and RACD based on the trans-cutting activity of cas9 combined with the nucleic acid amplification technology; therefore, the establishment of nucleic acid detection methods based on the trans-cutting activity of cas9 is also likely to be a hot spot for future research.

## 5. Conclusions

CRISPR/Cas technology has been proven to be highly suitable for nucleic acid detection. In recent years, there has been a growing application of CRISPR-based nucleic acid detection technologies in animal disease diagnosis. Primarily leveraging the CRISPR/Cas12 or CRISPR/Cas13 system, these technologies often employ fluorescence, electrochemical signal, or visualization methods to interpret outcomes. As a result, the diagnosis of some bacterial, viral, and parasitic animal diseases has become feasible. Remarkably, these methods achieve a detection limit as low as aM level. Compared with other animal disease detection methods, CRISPR/Cas-based nucleic acid detection technology combines the characteristics of rapidity, sensitivity, high specificity, and the ability for immediate detection, thus having great potential in animal disease diagnosis.

## Figures and Tables

**Table 1:** Classification of CRISPR/Cas systems and their characteristics.

Class	Type	Cas X	gRNA	Target	Collateral Cleavage	Features
Class1	Type I	Cas3		/	/	Multiple effector proteins
	Type IV	Csf1			/	
	Type III	Cas10			/	
Class2	Type II	Cas9	sgRNA	DNA/RNA	No	Single effector proteins
	Type V	Cas12a	crRNA	DNA	Yes	
		Cas12b	sgRNA	DNA	Yes	
		Cas14a	sgRNA	ssDNA	Yes	
	Type VI	Cas13a	crRNA	RNA	Yes	
		Cas13b	crRNA	RNA	Yes	

**Table 2:** Application of CRISPR/Cas nucleic acid-based assays in diagnosing animal infectious diseases in recent years.

Pathogen	Sample Type	Amplification	Cas	Output signal	Selectivity	Portability	LOD	Time	Ref.
*M. tuberculosis*	Genome	/	dCas9	Fluorescence	100%	No	1 copy/tube	/	[78]
*Salmonella*	Milk	PCR	Cas12a	Colorimetric/Electrical	100%	Yes	1 CFU/mL	1 h	[79]
*S. typhi*	Milk/meat	RPA	Cas12a	Electrical	100%	Yes	3–4 CFU/mL	55 min	[80]
*E. coli* O157:H7	Plasmid	RPA	Cas12a	Colorimetric	100%	Yes	/	40 min	[81]
*Bacillus anthracis*	Plasmid/blood	RPA	Cas12a	Fluorescence	100%	Yes	2 copy	40 min	[82]
*Brucella*	Milk/blood	RPA	Cas12a	Fluorescence/electrical	100%	Yes	2 copy/reaction	30 min	[83]
ZIKV	Serum	NABSA	Cas9	Colorimetric	100%	Yes	3 fM	3 h	[22]
ZIKV	Urine/blood/plasma/ Serum/saliva	RT-RPA	Cas13	Colorimetric	100%	Yes	1 copy/μL	<2 h	[29]
DENV	Serum/saliva	RT-RPA	Cas13	Colorimetric	100%	Yes	1 copy/μL	<2 h	[29]
ASFV	Genome/plasma	/	Cas12a	Fluorescence	100%	Yes	5.7 × 10^7^ copy/mL	2 h	[84]
ASFV	Blood/anal swabs	RPA	Cas12a	Colorimetric	100%	Yes	200 copy/reaction	40 min	[67]
PRRSV	Plasmid/tissue	RPA	Cas13a	Colorimetric	100%	Yes	172 copy/μL	/	[85]
PRRSV	Plasmid/spleen/lung	RT-RPA	Cas12a	Colorimetric	100%	Yes	1 copy/reaction	2 h	[86]
PEDV	Intestinal/fecal	RT-ERA	Cas12a	Colorimetric	100%	Yes	2 copy/reaction	1 h	[87]
PEDV	Plasmid/rectal swabs	RT-RAA	Cas12a	Colorimetric/Fluorescence	100%	Yes	1 × 10^2^ copy/reaction	1.5 h	[88]
PEDV/TGEV PDCoV/SADS-CoV	Plasmid	RT-LAMP	Cas12a	Colorimetric/Fluorescence	100%	Yes	1 copy/reaction	25 min	[89]
PCV3	Virus stocks/lymph node	ERA	Cas12a	Fluorescence/Electrical	100%	Yes	7 copy	1 h	[90]
CCHFV	Plasmid/virus stocks	RT-RPA	Cas13a	Fluorescence	100%	Yes	1 copy/μL	30–40 min	[91]
JEV	Brain	RT-LAMP	Cas12a	Colorimetric/Fluorescence	100%	Yes	8.97 or more copies	1 h	[92]
Mpox	Pseudo-viral particles	RPA	Cas12a	Colorimetric	100%	Yes	10.6 particles/μL	35 min	[93]
*L. donovani*	Spleen	RPA	Cas12	Fluorescence	100%	Yes	3.1 parasites	2.5 h	[94]
*R. tsutsugamushi*	Serum	RPA	dCas9	Electrical	100%	Yes	0.54 aM	25 min	[95]
*S. haematobium*	Urine	RPA	Cas12a	Fluorescence	100%	Yes	10 fg/reaction	2 h	[96]
*Plasmodium*	Plasmid//blood/ Anopheles dirus mosquitoes	RPA	Cas13a	Fluorescence	100%/94%	No	2.5–18.8 parasites	/	[97]
*Plasmodium*	Simulated samples/serum	RT-RPA	Cas12a	Colorimetric/Fluorescence	100%	Yes	2 parasites/μL	1 h	[98]
*To*	Soil	RAA	Cas12a	Colorimetric/Fluorescence	100%	Yes	10^−6^ nM	70 min	[99]
*To*	Plasmid/blood	RAA	Cas13a	Colorimetric	100%	Yes	1 × 10^−6^ ng/μL	2 h	[100]
*EHP*	Plasmid/hepatopancrea	RPA	Cas12a	Fluorescence	100%	Yes	50 copies	1 h	[101]

**Table 3:** Comparison of CRISPR-based detection technology with other detection methods.

Detection Method	Specificity	Sensitivity	Detection Time	Professional Equipment	Cost	Convenience	POCT
CRISPR/Cas	High	High	15–60 min	No	Medium	High	Yes
Pathogen detection	High	/	1–N d	Yes	Uncertain	low	No
Serologic detection	Medium	Medium	0.25–5 h	No	Low	High	Yes
PCR/qPCR	High	Medium–High	4–6 h	Yes	Medium–High	Low–Medium	No
Isothermal amplification technique	Medium	Medium	15–60 min	No	Low	High	Yes
Gene sequencing	Medium–High	High	1–15 d	Yes	High	Low	No

## Data Availability

No new data were created or analyzed in this study. Data sharing is not applicable to this article.
